# Real-World Outcomes of Neoadjuvant Dual Blockade in HER2-Positive Breast Cancer: The Role of Tumor Biology and pCR

**DOI:** 10.3390/jcm15062217

**Published:** 2026-03-14

**Authors:** Ayberk Bayramgil, Mehmet Haluk Yücel, Ezgi Turkoglu, Ali Kaan Guren, Fatih Kemik, Bedirhan Ulufer, Burçin Çakan Demirel, Anil Yildiz, Omer Sacli, Eda Ercin, Nazım Can Demircan, Oguzcan Kinikoglu, Sahin Lacin, Ahmet Bilici, Yunus Emre Altintas, Melike Ozcelik

**Affiliations:** 1 Department of Medical Oncology, Umraniye Training and Research Hospital, Istanbul 34764, Türkiye; dromersacli@gmail.com (O.S.); eda.ercin.ee@gmail.com (E.E.); yunusaltintas1688@gmail.com (Y.E.A.); drmelike.ozcelik@gmail.com (M.O.); 2 Department of Medical Oncology, Faculty of Medicine, Medipol University, Istanbul 34214, Türkiye; mhalukyucel@gmail.com (M.H.Y.); ahmetknower@yahoo.com (A.B.); 3 Department of Medical Oncology, Kartal Dr. Lütfi Kırdar City Hospital, Istanbul 34865, Türkiye; ezgiturk_90@hotmail.com (E.T.); ogokinikoglu@yahoo.com (O.K.); 4 Department of Medical Oncology, School of Medicine, Marmara University, Istanbul 34899, Türkiye; alikaanguren@gmail.com (A.K.G.); ncdemircan@gmail.com (N.C.D.); 5 Department of Medical Oncology, School of Medicine, Koç University, Istanbul 34010, Türkiye; kemikfatih41@gmail.com (F.K.); salacin@kuh.ku.edu.tr (S.L.); 6 Department of Medical Oncology, Faculty of Medicine, Istanbul University Institute of Oncology, Istanbul 34093, Türkiye; bedirhanulufer@hotmail.com; 7 Department of Medical Oncology, Bagcilar Training and Research Hospital, Istanbul 34200, Türkiye; burcin.cakandemirel@gmail.com; 8 Department of Medical Oncology, Basaksehir Cam and Sakura City Hospital, Istanbul 34303, Türkiye; anilyildiz@live.com

**Keywords:** breast cancer, HER2-positive breast cancer, hormone receptor expression, neoadjuvant therapy, pathological complete response

## Abstract

**Background/Objectives**: Neoadjuvant dual HER2 blockade is standard for HER2-positive breast cancer, yet response rates vary based on tumor biology. This multicenter study aimed to identify clinicopathological predictors of pathological complete response (pCR), focusing on quantitative hormone receptor (HR) expression and HER2 staining intensity, and to evaluate their impact on survival. **Methods**: This multicenter retrospective study included 290 female patients diagnosed with HER2-positive early or locally advanced breast cancer treated with neoadjuvant trastuzumab and pertuzumab-based regimens (anthracycline-based [AC-THP] or non-anthracycline [TCHP]) across six centers. HR expression was stratified into low (<50%) and high (≥50%) categories. Multivariable regression analyses identified predictors of pCR, Disease-Free Survival (DFS), and Overall Survival (OS). **Results**: The pCR rate was 51.4%. Multivariate analysis identified HR negativity (OR = 2.80; *p* < 0.001) and strong HER2 overexpression (IHC Score 3) (OR = 2.20; *p* = 0.037) as primary predictors. Uniquely, patients with low HR expression (<50%) achieved significantly higher pCR rates (65.9%) than strongly positive cases (36.6%; *p* = 0.001), biologically mimicking hormone-negative disease. The non-anthracycline TCHP regimen showed a strong trend toward superior efficacy (OR = 2.22; *p* = 0.054). pCR was the sole independent predictor of OS (HR = 0.134; *p* = 0.009). Crucially, adjusting for pCR unmasked hormone-negative status as a significant risk factor for recurrence (HR = 2.49; *p* = 0.028), highlighting its dual nature: high chemosensitivity but inherent biological aggression. **Conclusions**: “Strong” HER2 positivity and “weak” HR expression (<50%) are the primary determinants of pCR. pCR remains the strongest surrogate for survival, neutralizing initial risk factors. These findings support using quantitative biomarker thresholds for personalization and reinforce the efficacy of non-anthracycline regimens.

## 1. Introduction

Human epidermal growth factor receptor 2 (HER2)-positive breast cancer accounts for approximately 15–20% of all breast malignancies [1]. Although associated with aggressive disease and poor prognosis, outcomes have improved significantly with the introduction of HER2-targeted therapies, such as trastuzumab and pertuzumab [2]. Today, neoadjuvant systemic therapy is considered the standard treatment for locally advanced and high-risk early-stage HER2-positive breast cancer.

The primary goal of neoadjuvant therapy is to downstage the tumor and achieve a pathological complete response (pCR). pCR is widely recognized as a robust surrogate marker for long-term survival outcomes, particularly in HER2-positive disease. However, response rates are heterogeneous; hormone receptor (HR) status and HER2 staining intensity are known to influence chemosensitivity and treatment efficacy [3]. Additionally, although anthracycline-based regimens have traditionally been the cornerstone of treatment, concerns about cardiotoxicity have prompted increased use of non-anthracycline regimens, such as TCHP (docetaxel, carboplatin, trastuzumab, pertuzumab), which demonstrate comparable efficacy and a more favorable safety profile [4].

Recent real-world cohort studies and meta-analyses have highlighted the necessity of moving beyond a binary classification of HER2 and hormone receptor positivity. For example, varying degrees of HER2 amplification and quantitative levels of hormone receptor expression have been identified as potential modulators of neoadjuvant chemosensitivity. Nevertheless, real-world data that specifically examine the intersection of these quantitative biomarkers, particularly the distinction between IHC Score 3 and IHC 2+/FISH-amplified tumors in the context of dual blockade, remain limited and sometimes inconsistent. A more comprehensive understanding of these biological nuances is required to optimize patient selection and refine neoadjuvant treatment strategies.

This study aims to identify clinicopathological factors that predict pCR, with particular emphasis on quantitative hormone receptor expression and HER2 staining intensity. It also evaluates the prognostic impact of pCR and treatment regimens on disease-free and overall survival in a real-world cohort of patients with HER2-positive breast cancer.

## 2. Materials and Methods

### 2.1. Study Design and Patient Population

This multicenter retrospective study included 290 female patients diagnosed with HER2-positive early or locally advanced breast cancer who received neoadjuvant systemic therapy followed by curative surgery at six centers in Istanbul, Türkiye, between January 2015 and March 2025. The participating institutions were Ümraniye Training and Research Hospital, Kartal Dr. Lütfi Kırdar Training and Research Hospital, Medipol University Hospital, Koç University Hospital, Marmara University Training and Research Hospital, and Bağcılar Training and Research Hospital.

All patients were staged according to the TNM classification system of the American Joint Committee on Cancer (AJCC) Cancer Staging Manual (8th edition). Patients were included if they met the following criteria: histologically confirmed invasive breast carcinoma; confirmed HER2-positive status; received neoadjuvant chemotherapy containing dual anti-HER2 blockade (trastuzumab and pertuzumab); and had complete clinicopathological and follow-up data available. Patients with de novo metastatic disease (Stage IV), bilateral breast cancer, or those who did not receive neoadjuvant dual anti-HER2 blockade were excluded from the analysis. This study was reviewed and approved by the Ümraniye Training and Research Hospital Scientific Research Ethics Committee (Date: 23 October 2025, Approval No: B.10.1.TKH.4.34.H.GP.0.01/373). The study was conducted in accordance with the principles outlined in the Good Clinical Practice Guidelines and the Declaration of Helsinki.

### 2.2. Histopathological Evaluation and Receptor Status

Tumor biological characteristics were evaluated on pretreatment core biopsy specimens. Estrogen receptor (ER) and progesterone receptor (PR) status were determined using immunohistochemistry (IHC). HR positivity was defined as ≥1% nuclear staining for ER and/or PR. For this study, HR expression levels were further stratified into “low” (<50%) and “high” (≥50%) categories to evaluate the impact of expression intensity on treatment response. HER2 status was assigned according to the ASCO/CAP guidelines. Tumors with an IHC score of 3+ were defined as HER2-positive. Tumors with an equivocal IHC score (2+) were further evaluated using fluorescence in situ hybridization (FISH), and those with a HER2/CEP17 ratio ≥ 2.0 were confirmed as HER2-positive. Patients were categorized by HER2 detection: IHC Score 3 (strong overexpression) versus IHC Score 2/FISH-positive.

### 2.3. Treatment Protocols

Neoadjuvant systemic therapy was administered according to standard institutional protocols. The treatment regimens were categorized into two main groups:

1. Anthracycline-based Regimens (ddAC/THP): Patients received four cycles of Doxorubicin (60 mg/m^2^) combined with Cyclophosphamide (600 mg/m^2^) administered intravenously every 2 weeks (AC). This was followed by a taxane-based regimen consisting of Paclitaxel (80 mg/m^2^) administered weekly for 12 weeks. HER2-targeted therapy was initiated concurrently with the taxane component. Trastuzumab was administered as an 8 mg/kg loading dose followed by 6 mg/kg every 3 weeks, and Pertuzumab was given as an 840 mg loading dose followed by 420 mg every 3 weeks.
2. Non-anthracycline Regimen (TCHP): Patients received six cycles of Docetaxel (75 mg/m^2^) and Carboplatin (Area Under the Curve [AUC] 6), administered intravenously every 3 weeks. Dual HER2 blockade with Trastuzumab (8 mg/kg loading, 6 mg/kg maintenance) and Pertuzumab (840 mg loading, 420 mg maintenance) was administered concurrently throughout the six cycles.

The choice of neoadjuvant regimen in this retrospective multicenter cohort was influenced by institutional practice patterns, physician preference, patient-related factors, and reimbursement policies applicable in Türkiye during the study period. Dose adjustments or delays were determined by the treating physician based on treatment-related toxicities. After surgery, patients continued HER2-targeted therapy to complete a total duration of one year. Adjuvant radiotherapy and endocrine therapy were prescribed for eligible patients according to institutional standards.

### 2.4. Response and Survival Definitions

The primary endpoint of the study was pCR, defined as the complete absence of invasive tumor cells in both the breast and axillary lymph nodes (ypT0/is ypN0) upon histopathological examination of the surgical specimen. Secondary endpoints included Disease-Free Survival (DFS) and Overall Survival (OS). DFS was defined as the time from the date of surgery to the first documented locoregional recurrence, distant metastasis, or death from any cause. OS was defined as the time from the date of initial diagnosis to the date of death from any cause or the last follow-up visit.

### 2.5. Statistical Analysis

Statistical analyses were performed using SPSS Version 26.0 (IBM Corp., Armonk, NY, USA). Descriptive statistics were used to summarize baseline characteristics. Grammarly (Grammarly Inc., San Francisco, CA, USA; accessed February 2026) was used for English language editing and proofreading of this manuscript.

Categorical variables were compared using the Pearson Chi-square test or Fisher’s Exact test, as appropriate. Survival outcomes were estimated using the Kaplan–Meier method, and differences between groups were compared using the Log-Rank test. To identify independent predictors of pCR, univariate and multivariate binary logistic regression analyses were performed, and results were presented as Odds Ratios (OR) with 95% Confidence Intervals (CI). To identify independent prognostic factors for DFS and OS, univariate and multivariate Cox proportional hazards regression models were utilized, and results were expressed as Hazard Ratios (HR) with 95% CIs.

All statistical tests were two-sided, and *p*-values < 0.05 were considered statistically significant.

## 3. Results

A total of 290 patients with HER2-positive breast cancer were included in this analysis. Baseline patient and tumor characteristics are summarized in Table 1. The median age was 50 years (range, 24–85 years). Most patients presented with clinical Stage II disease (59.3%) and cT_2_ tumors (67.6%). Hormone receptor positivity was observed in 194 (66.9%) patients, and HER2 status was mainly determined by IHC Score 3 overexpression (86.2%).

Regarding neoadjuvant treatment, most patients (89.0%) received an anthracycline-based regimen (AC-THP), while 11.0% were given a non-anthracycline regimen (TCHP). After systemic neoadjuvant therapy, pCR was achieved in 149 patients (51.4%).

The median follow-up duration was 40.1 months. The 3-year DFS rate was 93.4%, and the 3-year OS rate was 96.8%. Based on the median follow-up of 40.1 months, the 3-year OS and DFS were assessed according to HER2 status, pCR status, hormone receptor status, and treatment type.

**Figure 1:**
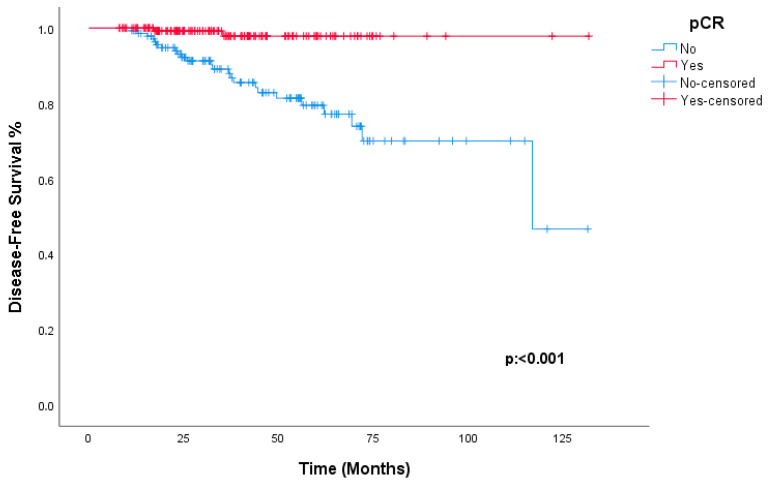
Kaplan–Meier estimates of disease-free survival (DFS) according to pathological complete response (pCR) status. Patients achieving pCR (red line) demonstrated significantly superior DFS compared to those with residual disease (blue line) (Log-Rank *p* < 0.001).

Analysis of DFS outcomes showed no statistically significant differences based on HER2 staining intensity (*p* = 0.924) or hormone receptor status (*p* = 0.598). Similarly, although the non-anthracycline (TCHP) arm exhibited an excellent profile with no recurrence events during follow-up (100% DFS), the difference compared to the anthracycline-based arm did not reach statistical significance (*p* = 0.257). The 3-year DFS rate was 97.9% in patients who achieved pCR, compared to 89.1% in those with residual disease (*p* < 0.001, Figure 1). Achieving pCR was linked to a 90.5% reduction in the risk of recurrence compared to residual disease (*p* = 0.001, HR = 0.095; 95% CI: 0.022–0.403).

**Figure 2:**
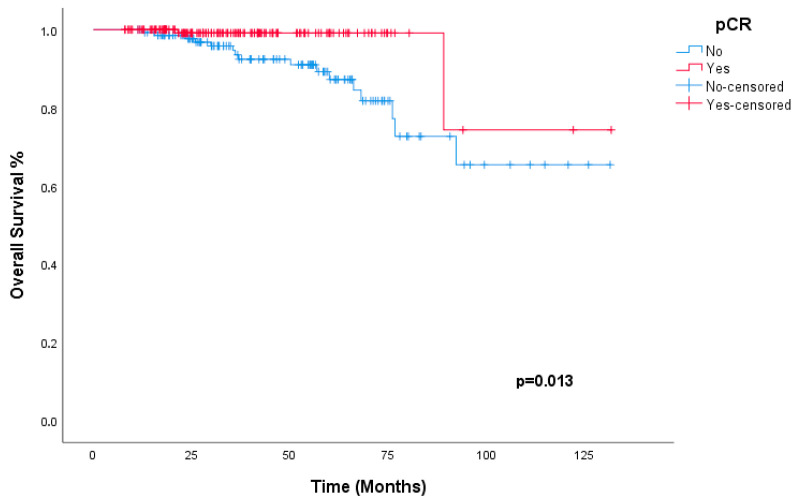
Kaplan–Meier estimates of overall survival (OS) according to pathological complete response (pCR) status. Patients achieving pCR (red line) demonstrated significantly superior OS compared to those with residual disease (blue line) (*p* = 0.013).

Consistent with the DFS findings, no statistically significant differences in OS were observed based on HER2 staining intensity (*p* = 0.679), hormone receptor status (*p* = 0.396), or treatment regimen (*p* = 0.558). The 3-year OS rate was 100% in patients who achieved pCR, compared to 94.7% in those with residual disease (Figure 2). Achieving pCR was associated with an 81.3% reduction in the risk of death compared to residual disease (*p* = 0.013, HR = 0.187; 95% CI: 0.042–0.820).

A multivariate Cox regression analysis was conducted to identify independent prognostic factors for DFS, including pCR and hormone receptor status as covariates. The analysis confirmed that both variables are statistically significant independent predictors of DFS. Patients who achieved a pCR experienced a 93.1% risk reduction compared to the residual disease group (*p* < 0.001, HR = 0.069; 95% CI: 0.016–0.301) (Table 2). Additionally, hormone receptor status became a significant independent risk factor after adjusting for pCR status (*p* = 0.028); hormone receptor negativity was associated with a 2.49-fold increased risk of recurrence (HR = 2.492; 95% CI: 1.105–5.623) (Table 2).

Multivariate analysis confirmed pCR status as the only significant independent predictor of OS (*p* = 0.009), providing an 86.6% risk reduction (HR = 0.134; 95% CI: 0.030–0.608). Additionally, hormone receptor negativity showed a strong trend toward poor prognosis, with a 2.54-fold increased risk of death (HR = 2.539), although this association was borderline significant (*p* = 0.056; 95% CI: 0.977–6.604) (Table 2).

Univariate analysis identified tumor biological characteristics and treatment regimens as significant predictors of pCR (Table 3). Regarding HER2 status, patients with strong immunohistochemical expression (IHC Score 3) achieved a significantly higher pCR rate compared to those with IHC Score 2/FISH+ status (54.8% vs. 30.0%; *p* = 0.004), with logistic regression confirming Score 3 status as a significant predictor (OR = 2.829; 95% CI: 1.376–5.816; *p* = 0.005). Similarly, a highly significant association was observed between hormone receptor status and treatment outcome; the pCR rate was substantially higher in hormone-negative patients compared to hormone-positive patients (68.8% vs. 42.8%; *p* < 0.001), and hormone negativity emerged as a strong predictor associated with a 2.94-fold increase in the odds of achieving pCR (OR = 2.942; 95% CI: 1.755–4.933; *p* < 0.001). Finally, regarding treatment protocols, patients receiving the non-anthracycline TCHP regimen showed a higher pCR rate compared to the standard AC-THP regimen (68.8% vs. 49.2%; *p* = 0.037), with the TCHP regimen significantly increasing the likelihood of pCR by 2.27 times (OR = 2.269; 95% CI: 1.034–4.982; *p* = 0.041). No statistically significant associations were found between pCR and other examined clinicopathological factors, including age, BMI, smoking or alcohol history, ECOG performance status, comorbidities, menopausal status, tumor location, histological subtype, histological grade, lymphovascular or perineural invasion, Ki-67 levels, and clinical tumor/nodal stage (all *p* > 0.05).

Among the 194 patients with hormone receptor-positive disease, a subgroup analysis was performed to assess how receptor expression levels (<50% vs. ≥50%) affected treatment response (Table 3). Patients with low HR expression (<50%) achieved a significantly higher pCR rate (65.9%) compared to those with high HR expression (≥50%), who had a pCR rate of only 36.6% (*p* = 0.001). Univariate logistic regression further clarified this relationship, showing that low HR expression was a strong predictor of response; patients with expression levels below 50% had 3.34 times higher odds of achieving pCR than those with higher expression (OR = 3.341; 95% CI: 1.619–6.894; *p* = 0.001).

To further assess pCR according to hormone receptor category, an exploratory subgroup analysis was performed within the HER2 subgroups. In the HER2 IHC 2+/FISH-positive subgroup, 6 patients (15.0%) were hormone receptor-negative, 2 (5.0%) had low hormone receptor expression (<50%), and 32 (80.0%) had high hormone receptor expression (≥50%). The corresponding pCR rates were 33.3%, 0.0%, and 31.3%, respectively. In the HER2 IHC 3+ subgroup, the corresponding pCR rates were 71.1%, 69.2%, and 38.0%. Detailed subgroup data are presented in Supplementary Table S1.

To assess the independent predictive value of the clinicopathological factors identified in the univariate analysis, a multivariate logistic regression model was developed that included hormone receptor status, HER2 staining intensity, and treatment regimen (Table 4). The analysis showed that hormone receptor status was the strongest independent predictor of pCR (*p* < 0.001). Specifically, patients with hormone receptor-negative tumors had nearly 2.8 times higher odds of achieving pCR compared to those with hormone-positive disease (OR = 2.797; 95% CI: 1.650–4.740). Additionally, HER2 overexpression level remained a significant independent prognostic factor (*p* = 0.037); patients with IHC Score 3 tumors had significantly higher odds of response compared to the Score 2/FISH+ group (OR = 2.204; 95% CI: 1.049–4.629). Regarding the treatment regimen, although the non-anthracycline TCHP protocol was associated with a clinically relevant increase in pCR probability (OR = 2.22) compared to the standard AC-based regimen, this association only reached borderline statistical significance in the multivariate model (*p* = 0.054).

## 4. Discussion

In this multicenter real-world study, we evaluated the predictors of pCR and survival outcomes in patients with HER2-positive breast cancer receiving dual HER2 blockade. Our findings confirm that achieving pCR is the most crucial factor for long-term survival, serving as the only independent predictor for OS in multivariate analysis. Furthermore, we demonstrated that tumor biology, specifically quantitative hormone receptor expression and HER2 staining intensity, plays a more decisive role in predicting treatment response than the choice of chemotherapy backbone.

The overall pCR rate of 51.4% observed in our study is consistent with the pivotal NeoSphere [2] (45.8%) and TRYPHAENA [4] (57–66%) trials, confirming the real-world efficacy of dual HER2 blockade. However, our analysis highlights the profound heterogeneity within HER2-positive disease. We found that hormone receptor status significantly affected response; patients with hormone-negative tumors had nearly three times the likelihood of achieving pCR compared to hormone-positive patients.

Uniquely, our subgroup analysis showed that even within the hormone-positive group, expression level is important: patients with low ER/PR expression (<50%) had significantly higher pCR rates (65.9%) than those with strongly hormone-positive tumors (36.6%). This indicates that “low-positive” tumors behave more like hormone-negative disease and might benefit more from cytotoxic therapy, a detail often missed in simple “positive/negative” classifications. The significant difference in pCR rates between the low (<50%) and high (≥50%) hormone receptor expression subgroups in our study supports the ‘endocrine masking’ hypothesis [5,6,7].

Another compelling finding is the impact of HER2 staining intensity. Patients with strong HER2 overexpression (IHC Score 3) had significantly higher odds of achieving pCR than those with equivocal HER2 status (IHC Score 2/FISH+). Our data suggest that the amount of HER2 on the cell surface may correlate with the magnitude of therapeutic inhibition. This observation supports the idea that IHC Score 3 tumors are a biologically distinct group with higher “oncogene addiction,” making them more responsive to HER2-targeted treatments [8,9].

The lower pCR rate in the HER2 IHC 2+/FISH-positive subgroup may be partly related to the predominance of strongly hormone receptor-positive tumors (80.0%). However, even hormone receptor-negative cases in this group show lower pCR rates. This suggests hormone receptor distribution alone is unlikely to fully explain the difference. The level of HER2 protein overexpression may influence sensitivity to neoadjuvant treatment. HER2 IHC 2+/FISH-positive tumors might represent a biologically distinct subset with less pronounced HER2-driven treatment responsiveness than HER2 IHC 3+ tumors. Nevertheless, the small number of HER2 IHC 2+/FISH-positive cases means this interpretation should be considered exploratory and hypothesis-generating, rather than definitive.

Our real-world data provides valuable insights into the ongoing debate regarding the necessity of anthracyclines in the era of dual HER2 blockade. The predominant use of the AC-THP regimen (89.0%) in our study reflects the national health insurance reimbursement policies and institutional practice patterns in Türkiye during the study period, where anthracycline-based sequential regimens were the primary accessible standard of care. The non-anthracycline TCHP regimen demonstrated a superior pCR rate (68.8%) compared to the standard anthracycline-based AC-THP regimen (49.2%) in univariate analysis. Although the small sample size in the TCHP arm warrants cautious interpretation, these results echo the findings of the TRAIN-2 study, which showed no significant difference in efficacy between anthracycline-containing and anthracycline-free regimens [10]. Given the well-documented cardiotoxicity risks associated with anthracyclines, our real-world data support the TCHP regimen as a highly effective and potentially safer alternative, especially for patients with cardiac comorbidities.

Perhaps the most definitive conclusion of our study is the prognostic value of pCR. Consistent with the strong association reported in the CTNeoBC meta-analysis [3], we found that pCR was associated with a substantial reduction in the risk of both recurrence (93.1%) and death (86.6%). Remarkably, in our multivariate survival analysis, conventional prognostic factors like clinical stage and histological grade lost their significance, leaving pCR as the only independent predictor of overall survival. Patients achieving pCR have an excellent prognosis regardless of their initial stage. In contrast, those with residual disease remain at high risk, highlighting the critical need for adjuvant salvage therapies such as T-DM1, as established in the KATHERINE trial [11].

A noteworthy finding in multivariate survival analysis was the emergence of hormone receptor status as a significant independent risk factor for recurrence (*p* = 0.028), despite its lack of significance in the univariate analysis. This phenomenon implies a ‘statistical suppression effect’ driven by the dual nature of hormone-negative disease. Biologically, hormone-negative/HER2-positive tumors are naturally more aggressive and tend to recur early more often than the hormone-positive subtype [7]. Conversely, these hormone-negative tumors show significantly higher chemosensitivity and are more likely to achieve pCR with neoadjuvant blockade [3,12]. In univariate analysis, the survival benefit from higher pCR rates counterbalances the inherent biological risk, effectively masking the impact of hormone status on survival. However, in the multivariate model, once the profound protective benefit of pCR is adjusted for, the underlying biological aggression associated with hormone negativity becomes apparent, revealing a 2.49-fold increased risk of recurrence. This underscores that, particularly in the absence of pCR, hormone-negative status confers a significantly worse prognosis [7].

Our study has several limitations due to its retrospective design. First, the non-randomized allocation of treatment regimens introduces potential selection bias; for instance, the TCHP arm was smaller, which may influence statistical power. Second, although the median follow-up of 40 months is sufficient to detect early relapses, longer follow-up is needed to evaluate late recurrences, especially in the hormone-positive subgroup. Third, the relatively modest sample size of 290 patients may restrict the ability to draw definitive conclusions in smaller sub-analyses. Nevertheless, the study’s multicenter design across diverse institutions, combined with strict inclusion criteria for a well-defined cohort receiving neoadjuvant dual HER2 blockade, accurately reflects real-world clinical practice. We believe these strengths mitigate the limitations associated with the sample size and significantly enhance the external validity and generalizability of our findings.

Future prospective studies and larger real-world registries are needed to better define optimal treatment de-escalation strategies for specific subgroups, particularly patients with HER2 IHC 2+/FISH-amplified and strongly hormone receptor-positive disease.

## 5. Conclusions

In summary, this study confirms that pCR is the strongest independent predictor of survival in HER2-positive breast cancer. We found that “strong” HER2 positivity (IHC Score 3) and “weak” hormone receptor expression (<50%) are the primary factors influencing pCR. These results indicate that neoadjuvant strategies should become more personalized, not only based on HER2 status but also on the quantitative levels of biomarkers, and they support the ongoing shift toward effective non-anthracycline regimens like TCHP.

## Figures and Tables

**Table 1:** Baseline Clinicopathological Characteristics of the Study Population (N = 290).

Characteristics	n (%)
Age (Years)	
Median, interval	50 (24–85)
<50	138 (47.6)
≥50	152 (52.4)
Sex	
Female	290 (100)
ECOG PS	
0	283 (97.6)
1	7 (2.4)
Menopausal Status	
Premenopausal	130 (44.8)
Perimenopausal	32 (11.0)
Postmenopausal	128 (44.1)
Tumor Size (T Stage)	
T1	50 (17.2)
T2	196 (67.6)
T3	30 (10.3)
T4	14 (4.8)
Nodal Status (N Stage)	
N0	41 (14.1)
N1	163 (56.2)
N2	53 (18.3)
N3	33 (11.4)
Histological Grade	
Grade X	54 (18.6)
Grade 1	6 (2.1)
Grade 2	102 (35.2)
Grade 3	128 (44.1)
Clinical Stage	
Stage I	7 (2.4)
Stage II	172 (59.3)
Stage III	111 (38.3)
Hormone Receptor Status	
Positive (ER+ and/or PR+)	194 (66.9)
Negative	96 (33.1)
Hormone Receptor Expression Level	
<50	41 (21.1)
≥50	153 (78.9)
HER2 Status	
IHC Score 3	250 (86.2)
IHC Score 2/FISH+	40 (13.8)
Neoadjuvant Regimen	
AC-THP (Anthracycline-based)	258 (89.0)
TCHP (Non-anthracycline)	32 (11.0)
pCR	
Achieved	149 (51.4)
No	141 (48.6)

**Table 2:** Multivariate Cox Proportional Hazards Regression Analysis of Independent Predictors for Disease-Free Survival and Overall Survival.

	DFS		OS	
Variable	HR (95% CI)	*p*-Value	HR (95% CI)	*p*-Value
pCR Status				
Achieved (vs. Residual Disease)	0.069 (0.016–0.301)	<0.001	0.134 (0.030–0.608)	0.009
Hormone Receptor Status				
Negative (vs. Positive)	2.492 (1.105–5.623)	0.028	2.539 (0.977–6.604)	0.056

Abbreviations: HR, Hazard Ratio; CI, Confidence Interval; pCR, Pathological Complete Response; DFS, Disease-Free Survival; OS, Overall Survival. Note: Reference categories are patients with residual disease (No pCR) and hormone receptor-positive status.

**Table 3:** Univariate Analysis of Clinicopathological Factors Predicting pCR.

Variable	Category	Total (N)	pCR n (%)	OR (95% CI)	*p*-Value
HER2 Status	Score 2/FISH+ (Ref)	40	12 (30.0)	1.00	
	Score 3	250	137 (54.8)	2.829 (1.376–5.816)	0.005
Hormone Status	Positive (Ref)	194	83 (42.8)	1.00	
	Negative	96	66 (68.8)	2.942 (1.755–4.933)	<0.001
Treatment Regimen	AC-THP (Ref)	258	127 (49.2)	1.00	
	TCHP	32	22 (68.8)	2.269 (1.034–4.982)	0.041
HR Expression Level *	High (≥50%) (Ref)	153	56 (36.6)	1.00	
	Low (<50%)	41	27 (65.9)	3.341 (1.619–6.894)	0.001

Abbreviations: pCR, Pathological Complete Response; OR, Odds Ratio; CI, Confidence Interval; Ref, Reference Category; AC-THP, Anthracycline-based regimen; TCHP, Non-anthracycline regimen; HR, Hormone Receptor. * Subgroup analysis performed only in hormone receptor-positive patients (N = 194). *p*-values were calculated using the Chi-square test and univariable logistic regression analysis.

**Table 4:** Multivariate Logistic Regression Analysis of Independent Predictors for pCR.

Variable	Category	*p*-Value	OR (95% CI)
Hormone Status	Negative (vs. Positive)	<0.001	2.797 (1.650–4.740)
HER2 Status	Score 3 (vs. Score 2)	0.037	2.204 (1.049–4.629)
Treatment Regimen	TCHP (vs. AC-based)	0.054	2.220 (0.986–5.001)

Abbreviations: pCR, Pathological Complete Response; OR, Odds Ratio; CI, Confidence Interval; Anthracycline-based regimen; TCHP, Non-anthracycline regimen. Note: Reference categories are Hormone Receptor Positive, HER2 Score 2/FISH+, and AC-THP treatment regimen.

## Data Availability

While the datasets analyzed in this study are not publicly accessible, they can be obtained from the corresponding author upon reasonable request.

## Supplementary Materials

The following supporting information can be downloaded at https://www.mdpi.com/article/10.3390/jcm15062217/s1: Supplementary Table S1. Distribution of hormone receptor categories and pCR rates according to HER2 subgroup.

## Abbreviations

The following abbreviations are used in this manuscript:

AC	Doxorubicin (Anthracycline) and Cyclophosphamide
AJCC	American Joint Committee on Cancer
ASCO	American Society of Clinical Oncology
AUC	Area Under the Curve
BMI	Body Mass Index
CAP	College of American Pathologists
CI	Confidence Interval
DFS	Disease-Free Survival
ECOG	Eastern Cooperative Oncology Group
ER	Estrogen Receptor
FISH	Fluorescence In Situ Hybridization
HER2	Human Epidermal Growth Factor Receptor 2
HR	Hormone Receptor (also Hazard Ratio in statistical context)
IHC	Immunohistochemistry
NCCN	National Comprehensive Cancer Network
OR	Odds Ratio
OS	Overall Survival
pCR	Pathological Complete Response
PR	Progesterone Receptor
TCHP	Docetaxel, Carboplatin, Trastuzumab, and Pertuzumab
T-DM1	Trastuzumab Emtansine
TNM	Tumor, Node, Metastasis classification
