# Supplementary material for: Real-World Outcomes of Neoadjuvant Dual Blockade in HER2-Positive Breast Cancer: The Role of Tumor Biology and pCR
Source: J Clin Med. 2026 Mar 14;15(6):2217. doi: 10.3390/jcm15062217 (PMC13026146; doi:10.3390/jcm15062217)
Supplement: Supplementary file 1 [file jcm-15-02217-s001.zip › jcm-4182006-supplementary.pdf]

Supplementary

Supplementary Table S1. Distribution of hormone receptor categories and pCR rates according to HER2 subgroup.

HER2 subgroup	HR category	Total n	pCR n	pCR %	Overall p value
**IHC 2+/FISH+**	Negative	6	2	33.3	0.634
**IHC 2+/FISH+**	Low (<50%)	2	0	0.0	
**IHC 2+/FISH+**	High (≥50%)	32	10	31.3	
**IHC 3+**	Negative	90	64	71.1	<0.001
**IHC 3+**	Low (<50%)	39	27	69.2	
**IHC 3+**	High (≥50%)	121	46	38.0	

p-values represent the association between hormone receptor category and pCR within each HER2 subgroup. Results for the HER2 IHC 2+/FISH-positive subgroup should be interpreted cautiously because of the limited sample size and sparse cell counts.
